# Thyroid Hormone and Neural Stem Cells: Repair Potential Following Brain and Spinal Cord Injury

**DOI:** 10.3389/fnins.2020.00875

**Published:** 2020-08-26

**Authors:** Pieter Vancamp, Lucile Butruille, Barbara A. Demeneix, Sylvie Remaud

**Affiliations:** Laboratory Molecular Physiology and Adaptation, CNRS UMR 7221, Muséum National d’Histoire Naturelle, Department Adaptations of Life, Paris, France

**Keywords:** brain and spinal cord injury, neurodegenerative disease, endogenous repair, neural stem cells, thyroid hormone, multiple sclerosis, stroke, Alzheimer’s disease

## Abstract

Neurodegenerative diseases are characterized by chronic neuronal and/or glial cell loss, while traumatic injury is often accompanied by the acute loss of both. Multipotent neural stem cells (NSCs) in the adult mammalian brain spontaneously proliferate, forming neuronal and glial progenitors that migrate toward lesion sites upon injury. However, they fail to replace neurons and glial cells due to molecular inhibition and the lack of pro-regenerative cues. A major challenge in regenerative biology therefore is to unveil signaling pathways that could override molecular brakes and boost endogenous repair. In physiological conditions, thyroid hormone (TH) acts on NSC commitment in the subventricular zone, and the subgranular zone, the two largest NSC niches in mammals, including humans. Here, we discuss whether TH could have beneficial actions in various pathological contexts too, by evaluating recent data obtained in mammalian models of multiple sclerosis (MS; loss of oligodendroglial cells), Alzheimer’s disease (loss of neuronal cells), stroke and spinal cord injury (neuroglial cell loss). So far, TH has shown promising effects as a stimulator of remyelination in MS models, while its role in NSC-mediated repair in other diseases remains elusive. Disentangling the spatiotemporal aspects of the injury-driven repair response as well as the molecular and cellular mechanisms by which TH acts, could unveil new ways to further exploit its pro-regenerative potential, while TH (ant)agonists with cell type-specific action could provide safer and more target-directed approaches that translate easier to clinical settings.

## Introduction

The human brain and spinal cord contain approximately 86 billion neurons and a similar number of non-neuronal cells (Azevedo et al., 2009). They form the central nervous system (CNS), a giant network controlling most of our body and mind. Injury to the CNS causes a loss of neuronal and/or glial cells, compromising neural network integrity. CNS insults include for example chronic autoimmune diseases that elicit neurodegenerative processes, or acute traumatic events such as stroke or a stabbing injury. Damage to the human CNS is usually irreversible due to the extremely limited capacity for self-repair, causing life-long intellectual or motor disability. The increasing incidence of neurodegenerative diseases in the population is currently the most important cause of morbidity and mortality, with millions of people affected (Heemels, 2016). This is particularly true for the elderly population with worldwide more than 10% of the plus 80 years old suffering from dementia in 2016, and accounting for 8.6% of the deaths of individuals aged 70 or more (Feigin et al., 2019).

The therapeutic goal for all types of CNS injury is to preserve or restore neural network integrity, and maintain or re-establish cognitive and motor functions, aiming at the highest possible life quality. One possibility is to harness better the endogenous capacity of the adult brain’s neural stem cells (NSCs) to generate new neurons and glial cells that could facilitate repair. Adult NSCs are multipotent, radial glia-derived cells that were set aside during development and have an unlimited self-renewal capacity *in vivo*. In the telencephalon of adult mammals, the majority of NSCs reside in two restricted niches, the subventricular zone (SVZ) lining the lateral ventricles and the subgranular zone (SGZ) of the hippocampus.

The SVZ contains the largest NSC population in adult mammals. In rodents, quiescent NSCs, or type B cells, juxtapose ependymal cells and have cerebrospinal fluid (CSF)-sensing cilia as well as processes enveloping blood vessels (Mirzadeh et al., 2008). They co-exist with activated, proliferating NSCs that successively give rise to transient amplifying proliferators (TAPs, or type C cells) and neuroblasts (type A cells), or less commonly, to oligodendrocyte precursor cells (OPCs) (Doetsch et al., 1999; Menn et al., 2006). In normal conditions, neuroblasts migrate via the rostro-caudal stream toward the olfactory bulbs where they differentiate into GABAergic and dopaminergic interneurons involved in olfaction (Lim and Alvarez-Buylla, 2016). OPCs migrate to adjacent brain regions such as the corpus callosum and can differentiate into myelinating oligodendrocytes (Menn et al., 2006) to facilitate myelin remodeling (Young et al., 2013; McKenzie et al., 2014). During aging, the NSC and TAP pools decrease, NSCs become quiescent and the neuro/glia ratio decreases sharply (Ahlenius et al., 2009; Capilla-Gonzalez et al., 2013; Daynac et al., 2016). In primates, including humans, the adult SVZ differs in some ways from that of rodents. A cell-devoid space separates ependymal cells and NSCs, and the fraction of quiescent NSCs is larger (Sanai et al., 2004; Quiñones-Hinojosa et al., 2006; Gil-Perotin et al., 2009). SVZ-neurogenesis in primates including humans drops sharply during the first months of life, only to continue at a very low rate thereafter, producing neuroblasts that predominantly migrate to the striatum with a so far unknown role (Sanai et al., 2004; Ernst et al., 2014). In contrast, a fairly large population of SVZ-OPCs exists in humans that can rapidly respond to injury (Nait-Oumesmar et al., 2007). While the SVZ neurogenic capacity declines even further during aging, the rate of SVZ-oligodendrogenesis seems to be constant throughout life (Weissleder et al., 2016).

In the hippocampus, radial glial-like NSCs or type 1 cells reside in the SGZ of the dentate gyrus. They can generate type 2a and 2b TAPs that, with the exception of a few astrocyte progenitors, exclusively form type 3 neuroblasts (Winocur et al., 2006). Post-mitotic neuroblasts differentiate into calretinin immature neurons and then into calbindin granule neurons during tangential and radial migration pathways in the dentate gyrus (Kempermann et al., 2015). They rewire hippocampal circuits involved in learning and memory throughout life, although hippocampal neurogenesis decreases with aging across species (Kozareva et al., 2019). The human hippocampus also contains NSCs that generate granule neurons and function in learning-dependent plasticity (Eriksson et al., 1998; Kozareva et al., 2019). In a recent study, Sorrells et al. (2018) did not detect *de novo* DCX-positive neuroblasts in the hippocampus of aged individuals. However, brain samples were fixed relatively late after death, which could have compromised tissue integrity and impeded antigen detection by immunohistochemistry (IHC). Furthermore, a substantial amount of brain samples was collected from patients with chronic epilepsy in whom neurogenesis might differ from basal levels (Lima and Gomes-Leal, 2019; Steiner et al., 2019). Others have used brain samples from deceased individuals without any record of neurological conditions, and fixed them more rapidly after death had occurred. Several independent studies detected thousands of DCX-positive neuroblasts being generated, demonstrating hippocampal neurogenesis throughout life, although the decreasing daily output during aging indicates a certain degree of plasticity loss (Boldrini et al., 2018; Moreno-Jiménez et al., 2019; Tobin et al., 2019).

Several types of CNS injury elicit SVZ- and SGZ-NSCs to actively proliferate and generate post-mitotic cells that can differentiate into mature neurons or glial cells, but this response never leads to functional restoration in humans (Picard-Riera et al., 2004; Faiz et al., 2015). Similarly, physical exercise and omega 3-enriched diet can amplify hippocampal neurogenesis and diminish cognitive decline in Alzheimer’s and Parkinson’s disease (Tincer et al., 2016; Morris et al., 2017), but never halt disease progression. Animal studies show that molecular inhibition and the lack of pro-regenerative cues constrains CNS repair in adults (Silver et al., 2015). While the competence to regenerate is limited to development in amniotes (Weil et al., 2008), phylogenetically primitive vertebrates such as fish and urodeles maintain spectacular regenerative capacities throughout their entire life, replacing entire body extremities and rebuilding lost brain connections from scratch (Genovese et al., 2013; Slack, 2017; Zambusi and Ninkovic, 2020). Extensive damage to the adult zebrafish telencephalon elicited an NSC-mediated response that fully repaired the injury after only a few weeks (Kishimoto et al., 2012). However, a highly similar transcriptome of adult zebrafish and mammalian NSCs suggests the latter also have a hidden or blocked regenerative potential (Lange et al., 2020). The challenge is to relieve the brakes on molecular inhibition and modulate pathways that promote regeneration, eliciting the repair capacity that is found in many non-mammalian vertebrates. Identifying such intrinsic and extrinsic signals that are capable of doing so, can open new avenues for enhancing endogenous CNS repair. Many factors have been identified over the years, including Notch and Wnt pathways (Lie et al., 2005; Aguirre et al., 2010), as well as choroid plexus-derived factors (Silva-Vargas et al., 2016), and hormones (Ponti et al., 2018). Here, we discuss thyroid hormone (TH) as a key signal in NSC commitment in the mammalian stem cell niches.

## The Potential of Thyroid Hormone as a Pro-Repair Cue

Thyroid hormone is a key endocrine signal conserved in all vertebrates, including humans, regulating many homeostatic processes such as growth, reproduction and energy metabolism. TH also regulates CNS development (Gothié et al., 2017) by influencing all neurodevelopmental processes, including cell cycle progression, fate choice, migration, differentiation, axo- and synaptogenesis, and myelination (Zoeller and Rovet, 2004; Moog et al., 2017; Krieger et al., 2019; Vancamp et al., 2020). Under pathophysiological conditions, TH acts on each of these processes, promoting regeneration in the adult fish brain that retained large numbers of NSCs (Grandel et al., 2006; Bhumika and Darras, 2014). On the contrary, in mammals, the regenerative potential is lost after a postnatal peak in THs, but THs continue to fine-tune exactly the same processes in the adult NSC niches as those occurring during neurodevelopment (Remaud et al., 2014; Kapoor et al., 2015; Gothié et al., 2020). Furthermore, our understanding of regulation of local TH action in NSCs has increased immensely over the past few years with TH, as well as novel TH analogs, emerging as interesting candidates for stem cell-based regenerative medicine.

### Thyroid Hormone Regulation in Adult Stem Cell Zones of the Healthy Brain

#### Regulation of Thyroid Hormone Metabolism

The hypothalamus-pituitary-thyroid (HPT) axis maintains systemic TH homeostasis by controlling the synthesis and secretion of the largely inactive prohormone thyroxine (T_4_), and to a lesser extent the biologically active T_3_, by the thyroid gland. The hypothalamic thyrotropin-releasing hormone (TRH) releases thyroid-stimulating hormone (TSH, or thyrotropin) from the pituitary to induce TH secretion into the bloodstream, the latter providing negative feedback on the HPT axis (Fekete and Lechan, 2014). Around 50% of the circulating T_4_ in humans is converted into T_3_ by the enzyme deiodinase type 1 (DIO1) in the liver (Bianco et al., 2002), after which both THs reach target tissues.

At the brain level, transmembrane transporters take up the lipophilic hormones T_4_ and T_3_. These comprise the monocarboxylate transporter 8 (MCT8) with a high affinity for T_4_ and T_3_, and the T_3_-transporter MCT10. Other secondary TH transporters such as the T_4_-selective organic anion transporting polypeptide 1C1 (OATP1C1) and the large amino acid transporters (LATs) also contribute to TH influx and efflux (Kinne et al., 2011; Groeneweg et al., 2020). THs first cross the endothelial cells of the blood-brain barrier (BBB), or the epithelial cells of the blood-cerebrospinal fluid barrier (BCSFB). In contrast to the rodent BBB, that expresses both MCT8 and OATP1C1 (Roberts et al., 2008; Wilpert et al., 2020), the human BBB almost completely lacks OATP1C1 (Roberts et al., 2008; Chan et al., 2014). At the BCSFB, T_4_ binds the TH-distributor protein transthyretin (TTR) releasing T_4_ into the CSF. Then, T_4_ circulates in the brain ventricles bordering regions such as the SVZ and spinal cord (Richardson et al., 2015). Once taken up by a neural cell, the enzyme DIO2 converts T_4_ into T_3_, whereas DIO3 inactivates T_4_ to reverse T_3_ (rT_3_) and T_3_ to T_2_, tightly modulating T_3_ availability (Luongo et al., 2019). DIO2 is particularly enriched in astrocytes that convert a considerable amount of T_4_ to T_3_ to supply to neurons and oligodendrocytes. The latter two express DIO3 to control intracellular T_3_ levels (Morte and Bernal, 2014).

T_3_ is considered as the more biologically active form of TH, as it can act on gene transcriptional activity in TH-targeted cell types by binding to nuclear TH receptors (TRs). Its availability and action in each cell or cell-type ultimately depends on specific expression patterns of the TH transporters, DIOs and receptors, and as such determines gene activity and biological processes. *THRA* and *THRB* genes encode the TR isoforms TRα1, TRα2, TRβ1 and TRβ2 in mammals (Bernal, 2007). Co-localization with IHC markers in TRα1-GFP mice showed that the majority of neurons and oligodendroglial cells express TRα1 (Wallis et al., 2010), whereas TRβ1 is enriched in astrocytes and some neuronal subpopulations. TRβ2 is more confined to cells in the hypothalamus and pituitary (Bernal, 2007). TRα2 is strongly and widely expressed in the brain, but its role as a dominant-negative receptor lacking affinity for T_3_ is still unclear (Guissouma et al., 2014). Genome-wide analyses identified hundreds of directly and indirectly T_3_-regulated genes in the mammalian brain (Chatonnet et al., 2015; Gil-Ibañez et al., 2017).

Of note, some non-canonical effects on neural cells have been described for other TH forms as well, but are restricted in their actions. For instance, T_4_ binding to the membrane α_ν_β_3_ integrin receptor promotes embryonic neural progenitor proliferation (Stenzel et al., 2014), and both T_4_ and rT_3_ can stimulate cerebellar granule cell migration and actin polymerization (Farwell et al., 2006). T_2_ has the potency to act on TRs (Köhrle, 2019), but a role in the CNS remains to be shown.

#### Thyroid Hormone Action in the Healthy SVZ

**FIGURE 1:**
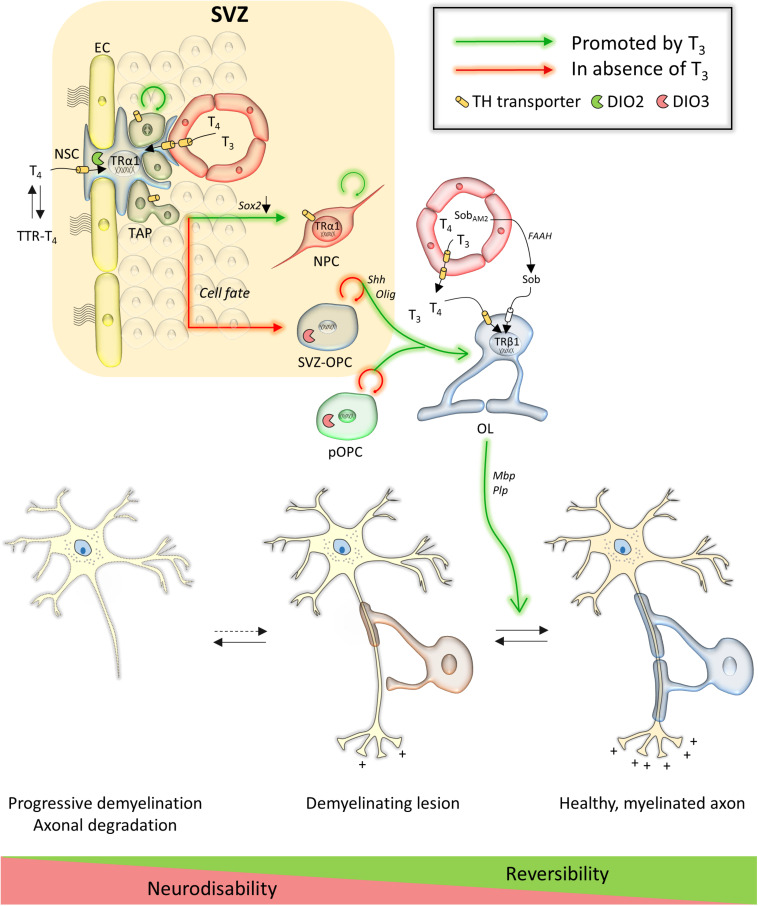
Overview of TH action in the subventricular zone and on oligodendroglial lineage cells and their contribution to repair in multiple sclerosis. Key genes are in italic next to arrows. Abbreviations: EC, endothelial cell; FAAH, fatty acid amide hydrolase; NPC, neural precursor cell; NSC, neural stem cell; OL, oligodendrocyte; Sob, sobetirome; Sob_AM2_, methyl amide-derivative of sobetirome; SVZ, subventricular zone; SVZ-OPC, SVZ-derived oligodendrocyte precursor cell; pOPC, parenchymal oligodendrocyte precursor cell; TAP, transient amplifying progenitor; TTR, transthyretin.

Data on the regulation of TH metabolism in NSCs almost exclusively originates from rodent studies. MCT8 and OATP1C1 mediate TH uptake at the endothelial cells of the rodent BBB as they do in other brain regions (Mayerl et al., 2014; Vatine et al., 2017), while TTR provides at least a part of T_4_ to SVZ-NSCs via the CSF (Vancamp et al., 2019; Alshehri et al., 2020). It is currently unknown which transporters facilitate TH uptake into SVZ-NSCs themselves. T_3_ is a master regulator of the NSC cycle and fate choice in the murine SVZ. Liganded TRα1 represses a gate-keeper of NSC identity, *Sox2*, and downregulates the cell cycle genes *c-Myc* and *Ccnd1*, facilitating TAP exit from the cell cycle and neuroblast commitment (López-Juárez et al., 2012; Remaud et al., 2017; Figure 1). In contrast, reduced T_3_ action in TAPs due to expression of the T_3_-inactivating DIO3, as well as TRα1 absence, allows *Egfr* expression. EGFR signaling promotes the generation of proliferating OPCs within the adult SVZ (Aguirre et al., 2007; Gonzalez-Perez and Alvarez-Buylla, 2011; López-Juárez et al., 2012; Remaud et al., 2017). If a T_3_-free window is required for OL progenitor specification, T_3_ also accelerates OPC cycle exit and differentiation through TRα1 (Durand and Raff, 2000; Billon, 2002), a process that also requires the nuclear receptor RXRγ (Baldassarro et al., 2019). Cell culture experiments indicate that liganded TRβ-induced expression of oligodendrogenesis-promoting genes such as *Klf9*, *Mbp*, and *Plp* allows for stepwise differentiation into mature and ultimately myelinating oligodendrocytes (Barres et al., 1994; Baas et al., 1997; Billon et al., 2001; Dugas et al., 2012).

#### Thyroid Hormone Action in the Healthy SGZ

Adult-onset hypothyroidism in rodents does not change the proliferative activity of type 1 SGZ-NSCs (Desouza et al., 2005; Kapoor et al., 2012; Sánchez-Huerta et al., 2016), nor does it change in *TR*α*1^–/–^* and *TR*α*2^–/–^* mice lacking and overexpressing TRα1, respectively (Kapoor et al., 2010), suggesting they are not responsive to TH. In contrast, thyroidectomized rats had less BrdU-positive and Ki67-positive hippocampal progenitor cells, an effect rescued by supplying T_4_ and T_3_ to the drinking water (Montero-Pedrazuela et al., 2006). Further studies have shown that SGZ-NSC self-renewal increases in *TR*β KO mice (Kapoor et al., 2011). Hence, either unliganded TRβ isoforms, or T_3_ acting through TRβ, repress SGZ-NSC turnover (Kapoor et al., 2015). The former hypothesis seems more likely as type 1 SGZ-NSCs and type 2 TAPs only express the low affinity TH transporters *Lat1* and *Lat2* (Mayerl et al., 2020), although definite proof is missing.

**FIGURE 2:**
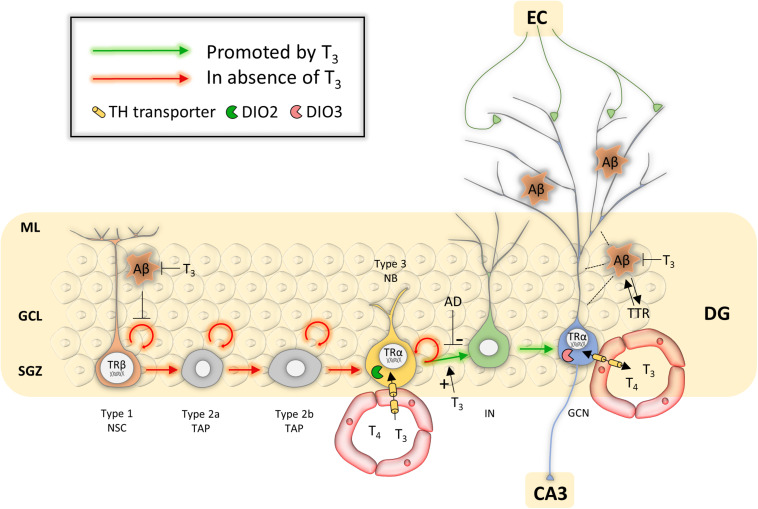
Overview of potential mechanisms of TH action during hippocampal neurogenesis in Alzheimer’s disease. Abbreviations: Aβ, amyloid β; AD, Alzheimer’s disease; DG, dentate gyrus; EC, entorhinal cortex; GCL, granule cell layer; GCN, granular cell neuron; IN, immature neuron; ML, molecular layer; NB, neuroblast; NSC, neural stem cell; SGZ, subgranular zone; TAP, transient amplifying progenitor; TTR, transthyretin.

T_3_-TRα1 action occurs from the post-mitotic stage, initiating type 2b TAPs to proceed to the differentiation phase and generate DCX-positive neuroblasts (Kapoor et al., 2010; Figure 2). The latter are in close proximity to the hippocampal blood vessel network, enabling BBB-mediated T_3_ uptake. qPCR on sorted SGZ cells and IHC showed that MCT8 is the only expressed TH transporter in type 3 neuroblasts. Deletion of *Mct8* either globally or in the hippocampal neurogenic lineage alone decreased expression of the cell cycle regulator P27KIP1, impairing neuroblast differentiation and granule cell genesis. The results indicate that MCT8 is the primary TH uptake route for neuroblasts during hippocampal neurogenesis (Mayerl et al., 2020). Additionally, TH determines granule cell survival by altering the expression of pro- and anti-apoptotic factors under the influence of TRs, with a higher expression of TRβ compared to TRα1 and TRα2 (Guadaño-Ferraz et al., 2003; Desouza et al., 2005; Kapoor et al., 2010, 2011; Mayerl et al., 2020). *Dio2* KO mice have a decreased hippocampal T_3_ content and display emotional alterations (Bárez-López and Guadaño-Ferraz, 2017), suggesting local DIO2 is another key component for generating sufficient T_3_ and stimulating cell differentiation and maturation, though its cellular expression pattern remains to be unraveled. Immature and mature granule cells also contained detectable levels of DIO3, LAT2 and MCT10 (Mayerl et al., 2020), but their functional roles remain unknown.

### Patient Studies Linking Thyroid Hormone Levels With CNS Injury Outcome

A last line of evidence showing that TH could aid in CNS repair comes from epidemiological studies. Such studies indicate that low TH levels can predispose to neurodegenerative diseases, and can also have unfavorable outcomes in terms of morbidity and mortality following CNS injury. Mild thyroid dysfunction, a common condition in the elderly (Biondi et al., 2019), or abnormally low TH levels, both increase the risk for developing neurodegenerative diseases, or correlate with worsened outcomes. For example, an inverse relation between free T_3_ levels and the risk for developing Alzheimer’s disease (AD) was demonstrated in a 302 patient cohort with mild cognitive impairment at the beginning of the study (Quinlan et al., 2019). Similarly, lower free T_3_ levels in pre-stroke individuals correlated with increased stroke severity, disability and mortality rates (Jiang et al., 2017; Lamba et al., 2018).

Unrelated to the patient’s endocrinological history, CNS disease or injury is often also followed by a physiological drop in T_3_ levels. Depressed serum T_3_ levels have been observed years ago in many patients with chronic spinal cord injury (SCI) (e.g., Chen et al., 2012), and between 32–62% of patients that suffered from a stroke had lower than normal T_3_ levels. Low circulating T_3_ levels were also found in multiple sclerosis (MS) patients (Zych-Twardowska and Wajgt, 2001). This is known as low T_3_ syndrome or *euthyroid sick syndrome* (ESS or Non-thyroidal Illness Syndrome), an adaptive body response that reduces metabolic and energetic needs during recovery (Mourouzis et al., 2013; Bunevicius et al., 2015). However, while being beneficial at first, sustained low T_3_ levels in the secondary phase of the disease provoke unwanted complications and poorer rehabilitation (Bunevicius et al., 2015; Fliers et al., 2015). Anticipating ESS by administering THs prior to major surgery has led to better recovery (Liu et al., 2014; Zhang et al., 2018). The correlation between TH status and outcome in unrelated CNS diseases implies that TH impacts repair processes in all of them, though these studies do not reveal the mechanisms implicated.

Altogether, TH seems a promising candidate for regenerative medicine because it (i) profoundly impacts neurodevelopmental plasticity, (ii) contributes to neural repair in primitive vertebrate species throughout life, (iii) controls NSC behavior in the healthy adult rodent SVZ and SGZ, (iv) finally, TH status plays a key role in CNS disease and injury outcome. Different research groups have therefore investigated how THs could promote endogenous repair in CNS injury or disease by acting on NSCs. We elaborate on results obtained in canonical CNS disorders that, despite different etiologies, could all benefit from cell replacement and neural network repair. These include the chronic neurodegenerative diseases MS (loss of oligodendrocytes) and AD (loss of neuronal cells), as well as acute traumas such as stroke and SCI, in which entire neural networks vanish in a matter of minutes. Each of them has unique features with regard to cell type loss, affected tissue/structure, location, and temporal disease patterns, posing different problems for stem cell-based approaches. Directing cell fate choice to generate the whole panel of cell types in correct ratios is paramount but injury-dependent. Newly generated precursor cells will also have to migrate enormous distances to the site of injury and incorporate existing but damaged neural networks, or re-join previously connected networks. An additional challenge is that many CNS insults predominantly affect the elderly in which TH homeostasis is altered (Bowers et al., 2013) and the endogenous repair potential in the stem cell zones is probably less effective in producing the various cell types as compared to young individuals (Capilla-Gonzalez et al., 2015). We discuss the scientific evidence that so far supports a role for TH in NSC-mediated repair in the above-mentioned CNS insults, and provide future perspectives on how to tackle current bottlenecks.

## The Repair Potential of Thyroid Hormone in Multiple Sclerosis

Multiple sclerosis is a chronic demyelinating CNS disease with around 2.2 million cases worldwide in 2016, a 10% increase compared to 1990. Prevalence is highest in 55–64 year-olds, but the disease is often diagnosed in young adults around the age of 30. Recurring episodes of autoimmune-mediated oligodendrocyte apoptosis cause progressive, heterogeneous myelin degradation (Haider et al., 2016) and neurological disability, affecting life quality and decreasing average life expectancy by several years. While current treatments primarily focus on attenuating the relapsing immune reactions, reversing demyelination and restoring neurological functions also hold promise as definitive remedies. Repair comprises regenerating lost oligodendrocytes to remyelinate naked axons and protect them from a hostile microenvironment (Calzà et al., 2010). However, while a fairly large population of parenchymal OPCs (pOPCs), generated during development, persists in the adult brain, most of them fail to differentiate into new myelinating oligodendrocytes (Wolswijk, 1998; Chang et al., 2000). The few pOPCs that do, wrap axons in thinner than normal myelin sheaths, a hallmark visible as shadow plaques on brain magnetic resonance imaging (MRI) (Patrikios et al., 2006). Exhaustion of the resident pOPC pool and failed migration to distant sites of injury further contribute to progressive neurological deterioration in the majority of MS patients (Hartley et al., 2014; Dulamea, 2017).

The adult SVZ could reinforce endogenous repair in MS, providing a source of newly generated OPCs. Furthermore, and in contrast to pOPCs, SVZ-derived OPCs generate myelin sheaths with a normal thickness (Xing et al., 2014; Brousse et al., 2015; Remaud et al., 2017). Brain examinations of deceased patients showed activated generation of SVZ-OPCs that successfully migrated to lesion sites within the corpus callosum (Nait-Oumesmar et al., 2007). Boosting this inherent repair system could thus improve functional repair by (i) amplifying the generation of OPCs from cycling SVZ-NSCs and promoting glial cell fate, (ii) stimulating OPC migration to lesion sites and, (iii) promoting their differentiation into myelinating oligodendrocytes.

### Thyroid Hormone Promotes Remyelination in Animal Models of Multiple Sclerosis

As a key signal that controls SVZ-NSC proliferation (Lemkine et al., 2005) and triggers OPC differentiation in the healthy adult brain (Dugas et al., 2012), it has been tested extensively if TH could improve remyelination in mammalian models of cuprizone-induced demyelination (Table 1). Cuprizone is a widely used gliotoxin inducing oligodendrocyte death and demyelination in brain regions, notably in the corpus callosum, provoking neurobehavioral deficits such as gait problems and sensorimotor impairments that mimic several symptoms observed in MS patients (Ludwin, 1978; Franco-Pons et al., 2007; Praet et al., 2014). Adult rats were fed with 0.6% cuprizone for 2 weeks to induce demyelination and received three subcutaneous T_3_ injections every 2 days during the first week of remyelination. Examination directly after the treatment showed a smaller pool of Nestin-positive progenitors and more OLIG-positive OPCs in the SVZ, suggesting that systemic T_3_ treatment stimulated SVZ-OPC cycle exit. The authors observed an upregulation of TRα by IHC in the treated and untreated cuprizone groups, although the TR was surprisingly absent in controls (Franco et al., 2008), conflicting with previous data (Lemkine et al., 2005). TRβ expression in the SVZ also increased following demyelination and T_3_ injection, whereas it was undetectable in healthy conditions or after cuprizone treatment alone (Lemkine et al., 2005; Franco et al., 2008). T_3_ is a potent inducer of *THRB* gene transcription (Baas et al., 1998), and increased TRβ1 protein levels in cultured rat OPCs as well as *in vivo* during postnatal development (Baxi et al., 2014). This suggests that T_3_-dependent upregulation of this TR isoform could precede OPC migration. Increased numbers of mature myelinating oligodendrocytes in the corpus callosum positive for O4, MBP, PLP and CC1 further indicated that T_3_ treatment applied after a cuprizone diet, improves remyelination (Franco et al., 2008). However, the 1-week treatment might not have been sufficient to reach control levels. While the origin of the *de novo* oligodendrocytes, derived from either SVZ-OPCs or pOPCs, was not determined, tracing experiments demonstrated that SVZ-OPCs successfully migrate toward the demyelinated corpus callosum adjacent to the SVZ (Xing et al., 2014; Brousse et al., 2015; Remaud et al., 2017) where they subsequently differentiate into immature and mature, myelin sheath-forming oligodendrocytes (Fernandez et al., 2004; Franco et al., 2008; Zhang et al., 2015; El-Tahry et al., 2016) (Figure 1).

**TABLE 1:** Summary of thyroid hormone effects in MS models.

**Study**	**Species**	**Model**	**Treatment**	**Effects**
(Calza et al., 2002)	♀ rats	EAE	s.c. T_4_	Proliferating cells SVZ + SC ↓ OPC + Mature OL markers ↑ NGF content ↑
(Fernandez et al., 2004)	♀ rats	EAE	s.c. T_4_	OPC + Mature OL markers ↑ Myelin sheath reassembly ↑ Relapse severity ↓ Axon morphology ↑
(Franco et al., 2008)	rats	CPZ	s.c. T_3_	Proliferating cells SVZ ↓ OPC cycle exit ↑ SVZ TRβ expression ↑ OL markers CC ↑ Remyelination of the CC
(Harsan et al., 2008)	♀ mice	CPZ	i.p. T_3_	Proliferating cells SVZ ↑ OPCs & Neuroblasts ↑ Myelinated axons ↑ MRI WM status CC & Cb ↑ Motor performance ↑
(Fernández et al., 2009)	Neurospheres derived from EAE rats	/	T_3_	Proliferation ↓ OPC genesis ↑ OL maturation ↑
(D’Intino et al., 2011)	♂ + ♀ marmosets	EAE	s.c. T_3_	OPC + Mature OL markers ↑ Disability score ↓ Preservation myelin sheaths ↑ Inflammation ↓ Normalization tissue TH metabolism
(Dell’Acqua et al., 2012)	♀ rats	EAE	s.c. T_3_	Disability score ↓ Neurophysiological parameters ↑ Remyelination ↑ Nerve impulse propagation ↑ Neurofilament levels normalized TRα1 + TRβ expression normalized
(Castelo-Branco et al., 2014)	♂ + ♀ rats	EAE	s.c. T_4_ + VA	Myelin loss ↓ OPC + Mature OL markers = Inflammation ↓ Clinical signs ↑
(Zhang et al., 2015)	mice	CPZ	i.p. T_3_	OPC proliferation and differentiation ↑ Remyelination in the CC Myelinated axons ↑ Myelin thickness/g-ratio = MRI WM status ↑ Clinical signs ↑
(El-Tahry et al., 2016)	♂ rats	CPZ	s.c. T_3_	Mature OL markers ↑ Myelinated axons ↑ Remyelination in the CC
(Fernández et al., 2016)	OPC-enriched cell culture	Cytokine exposure	IOP + T_4_ + T_3_	OPC differentiation recovery *Klf9* gene expression normalized
(Remaud et al., 2017)	♂ mice	CPZ	MMI during CPZ exposure followed by T_4_ + T_3_	SVZ-OPCs ↑ g-ratio ↓ (normal myelin thickness) Functional remyelination in the CC
(Shultz et al., 2017)	♀ rats	CCI	Local hydroxygel-based T_3_ delivery	Oligodendrogenesis *in vitro* ↑ OL maturation *in vitro* ↑
(Hartley et al., 2019)	OPCs from P7 rats		T_3_ sobetirome	Sobetirome mimics T_3_ action *Klf9*, *Mbp*, *Hr* expression ↑
	♂ mice	CPZ	i.p. T_3_ or i.p. sobetirome	Remyelination in the CC Mature OLs ↑ Myelinated axons ↑
	♂ mice	Lysolecithin	i.p. T_3_ or i.p. sobetirome	
	♂ + ♀ *iCKO-Myrf* mice	/	T_4_ + T_3_ or PTU sobetirome or Sob-AM_2_ chow	Both had adverse effects on long-term remyelination T_4_ + T_3_ OPCs + OLs ↓ OL maturation ↑ Myelinated axons ↑ Remyelination ↑ WM MRI status ↑ Motor performance ↑

*Abbreviations: Cb, cerebellum; CC, corpus callosum; CCI, cervical contusion injury; CPZ, cuprizone; EAE, experimental autoimmune encephalomyelitis; Hr, hairless; IOP, iopanoic acid; i.p., intraperitoneal; Klf9, Krüppel-like factor 9; MBP, myelin basic protein; MMI, methimazole; MRI, magnetic resonance imaging; NGF, neural growth factor; NSC, neural stem cell; OL, oligodendrocyte; OPC, oligodendrocyte precursor cell; PTU, propylthiouracil; s.c., subcutaneous; SC, spinal cord; SVZ, subventricular zone; TH, thyroid hormone; TR, thyroid hormone receptor; VA, valproic acid; WM, white matter.*

In another study, mice were fed a 0.2% cuprizone diet for 12 weeks, reaching a profound demyelination (Harsan et al., 2008). Intraperitoneal T_3_ injections were given daily during the 3-week recovery period after withdrawal of the cuprizone diet, and mice were evaluated up until 9 weeks thereafter. At that time, remyelination in the corpus callosum and cerebellum had improved as seen on MRI and IHC for MBP, a key TR-target gene (Jeannin et al., 1998), and numbers of myelinated axons almost reached control levels (Harsan et al., 2008). Thus, a brief TH treatment after demyelination can have long-lasting beneficial effects on remyelination. Furthermore, T_3_ increased numbers of migrating PSA-NCAM-positive neuroblasts in the SVZ, suggesting a neurogenic response as well. Motor performance on rotarod tests gradually improved as compared to mice that did not receive exogenous T_3_ (Harsan et al., 2008). Similar behavioral improvements were observed in mice that received daily intraperitoneal T_3_ injections for 2 weeks after a 0.2% cuprizone diet for 6 weeks (Zhang et al., 2015). Numbers of CC1-positive mature oligodendrocytes, MBP immunoreactivity and the proportion of myelinated axons increased in the corpus callosum as shown by western blotting, IHC and electron microscopy. As expected, all these effects were abolished when T_3_ was replaced by the thyroid-blocking agent propylthiouracil during the remission period (Zhang et al., 2015).

The potential of TH to promote OPC differentiation and maturation was also investigated in rats and marmosets in which experimental autoimmune encephalomyelitis (EAE) elicited spontaneous neuroglial apoptosis and axonal degeneration in the CNS, causing long-term sensorimotor disability (Constantinescu et al., 2011) (Table 1). A pulse of T_3_ every other day increased proliferation in NSC cultures prepared from the SVZ of EAE Lewis rats, and stimulated OPC differentiation and maturation into myelinating oligodendrocytes. T_3_ had similar pro-oligodendrogenic effects in OPC cultures grown from healthy SVZ-NSCs that during *in vitro* differentiation were simultaneously exposed to a mix of cytokines that mimic inflammatory conditions (Fernández et al., 2009, 2016). These properties were also tested *in vivo*. Adult rats received three subcutaneous T_4_ injections every other day during the acute EAE phase, when animals suffer from a severe force deficit (Calza et al., 2002). The strongly increased proliferation of SVZ-NSCs and uncommitted progenitors (Calzà et al., 1998) returned to basal levels in EAE animals treated with T_4_. This contrasts with the increased proliferative activity following T_3_ treatment *in vitro* (Fernández et al., 2009), and could be a consequence of different treatment protocols as well as the influence of factors in the complex *in vivo* environment. IHC analysis in the spinal cord showed less BrdU-positive progenitors and more A2B5-, O4- and MBP-positive committed OPCs, pre-oligodendrocytes and differentiated oligodendrocytes, respectively (Calza et al., 2002). Using the same protocol but now on Dark Agouti rats that have a more pronounced demyelination phenotype than Lewis rats, pulsed T_4_ administration at 2 and 3 weeks after immunization also promoted spinal cord remyelination. PDGFRα and MBP protein levels increased, and myelin sheaths thickened in the chronic recovery phase (Fernandez et al., 2004). Similarly, a 3-week pulsed T_3_ treatment from 10 days post-immunization increased the fluoromyelin-positive area in the spinal cord of EAE rats and improved neurobehavioral functions. Twenty-four hours after the first T_3_ pulse, the increase in *TR*α*1* and *TR*β expression following EAE was prevented, suggesting rapid effects on gene transcription (Dell’Acqua et al., 2012). Similarly, a short 3-day treatment with T_3_, 2 weeks prior to and a second time 4 weeks after inducing EAE in marmosets (*Callithrix Jacchus*), ameliorated the clinical disability score and increased the expression of oligodendrocyte markers in the spinal cord (D’Intino et al., 2011).

Irrespective of the different treatments protocols and the fact that many observations were made in the spinal cord, these studies strongly suggest that TH treatment triggers NSC and progenitor cell-cycle exit and promotes OPC differentiation by stimulating several genes in post-mitotic OLs (Figure 1), and that in all animal models of demyelination (Table 1). The fact that T_4_ and T_3_ are both capable to mediate these effects on gene expression (Fernández et al., 2016) suggests an important role for DIO2-mediated conversion T_4_ to T_3_ either by OPCs, or by astrocytes that are known to provide T_3_ to neurons in other brain regions as well (Morte and Bernal, 2014). T_4_ and T_3_ additionally had anti-inflammatory and neuroprotective effects on axonal morphology by increasing *Ngf* expression (Calza et al., 2002; D’Intino et al., 2011; Castelo-Branco et al., 2014; Fernández et al., 2016).

A problem that would arise in clinical settings is that TH provokes adverse side effects on peripheral organs such as bone, muscle and heart. In order to circumvent that issue, Hartley and co-workers (Hartley et al., 2019) used the pro-drug Sob-AM_2_ that is converted into the TRβ agonist sobetirome by the CNS-specific enzyme fatty acid amide hydrolase (Meinig et al., 2019). Sobetirome stimulated differentiation of rodent and human cultured OPCs, and accelerated developmental myelination in mice (Baxi et al., 2014). Applying sobetirome to cultured rat OPCs demonstrated its capacity to induce expression of T_3_-responsive pro-oligodendrogenic genes (e.g., *Mbp*, *Klf9*, *Hr*). The potential of sobetirome and Sob-AM_2_ to promote remyelination was then tested in three different mouse models (Hartley et al., 2019): (i) mice that received lysolecithin injections to focally demyelinate the corpus callosum (Blakemore and Franklin, 2008) ± daily sobetirome injections for 5 days thereafter, (ii) mice fed a 12-week 0.2% cuprizone diet ± daily sobetirome injections for 3 weeks thereafter, and (iii) *iCKO-Myrf* mice in which mature oligodendrocytes death leads to gradual demyelination with preservation of the OPC pool (Koenning et al., 2012) ± daily sobetirome or Sob-AM_2_ chow for 22 weeks, starting 2 weeks after tamoxifen treatment was induced. All treatments were performed in parallel with T_3_. In all models, sobetirome increased the MBP signal, PDGFRα-positive mature oligodendrocytes, myelinated axons, myelin content in the corpus callosum, and rotarod motor performance (Table 1). Furthermore, Sob-AM_2_ successfully induced long-term remyelination in the *iCKO-Myrf* mouse model without any measurable side-effects (Hartley et al., 2019), highlighting the potential of a receptor isoform-specific TH analog as a powerful targeted approach (Figure 1).

### Current Bottlenecks in Thyroid Hormone-Mediated Repair in Multiple Sclerosis

As discussed above, THs have beneficial and reproducible effects on stimulating spinal cord and SVZ-derived OPCs as well as pOPCs to differentiate and remyelinate in various models of MS. However, these exciting results also raised new questions that will need to be addressed in future studies. A first issue relates to the size of the SVZ-OPC pool. While SVZ-OPCs contributed strongly to remyelination in the mouse brain after a relatively short 5-week long cuprizone exposure, prolonged demyelination following a 12-week exposure, reflecting the human situation of MS more accurately, depleted the SVZ-OPC pool (Brousse et al., 2015). As TH stimulates OPC differentiation, protracted TH treatment may even accelerate exhaustion of the SVZ-OPC pool, stalling remyelination in the early, reversible phase of the disease. Prolonged T_3_ treatment in different mouse models of MS abrogated the increase in proliferating SVZ-NSCs and –OPCs that was seen in non-treated animals, and prematurely ceased OPC proliferation and oligodendrocyte formation (Franco et al., 2008; Hartley et al., 2019). Since glial lineage commitment occurs in the absence of TH signaling (Remaud et al., 2017), transient methimazole-induced hypothyroidism during the last 4 weeks of a 6-week cuprizone diet in adult mice, when new OPCs are proliferating to participate to myelin repair (Picard-Riera et al., 2002), enhanced SVZ-OPC generation. When followed by three T_4_ and T_3_ pulses after cuprizone withdrawal, remyelination in the corpus callosum accelerated compared to animals that were euthyroid during demyelination (Remaud et al., 2017). In addition, in normal conditions, downregulating *Dio3* expression mainly in EGFR-positive and PDGFRα-positive OPCs by transfecting SVZ-cells with a short hairpin *Dio3* construct, reduced global cell proliferation by 50% and OLIG2-positive cells by 30% (Remaud et al., 2017). This proves that reduced T_3_ signaling favors SVZ-OPC generation in physiological conditions, and also after a demyelinating lesion. It are these divergent results that relate to different treatments protocols (e.g., cuprizone diet, start of TH treatment, duration, and dose) in pre-clinical MS studies that will ultimately allow distilling an optimal treatment scenario. Ideally, TH treatment would be finely tuned to keep a steady balance between NSC-derived OPC generation, proliferation and OPC differentiation, promoting constant and long-term remyelination (Figure 1).

Another question is whether SVZ-OPCs can efficiently migrate to lesion sites. Demyelinating lesions are scattered throughout the gray and white matter of the brain, in regions distant from the SVZ, such as the cortex, cerebellum and brain stem (e.g., Kutzelnigg et al., 2005, 2007) as well as the spinal cord (Gilmore et al., 2006). Lesions in these CNS regions are most-likely responsible for motor disability in MS patients and hence are the prime targets for remyelination (Filippi et al., 2019). SVZ-OPCs migrate efficiently to the adjacent corpus callosum in demyelinating conditions in a matter of days (Xing et al., 2014; Brousse et al., 2015; Remaud et al., 2017). In contrast, colonization and remyelination by newly generated SVZ-OPCs after a 6-week cuprizone treatment in mice was slower and less complete in the cingulate cortex (± 60% vs. controls) and hippocampus (± 90% vs. controls) 6 weeks after demyelination, and mature oligodendrocyte numbers remained very low (Baxi et al., 2017). Accordingly, in MS patients, remyelination was successful in regions in close proximity to the SVZ, while shadow plaques remained in the cortex and spinal cord (Patrikios et al., 2006). Several reports showed that TH treatment improved white matter status in the murine cerebellum and cortex (Harsan et al., 2008; Silvestroff et al., 2012; Hartley et al., 2019). While the exact origin of the OPCs responsible for remyelination in these brain regions was not determined, selective *Olig2* overexpression in SVZ-progenitors of postnatal mice showed that the OPC progeny was capable of migrating to the cortex (Maire et al., 2010).

It seems unlikely that SVZ-NSCs or –OPCs can abundantly migrate to the spinal cord. However, here, ependymal cells (ECs) surrounding the central canal in the spinal cord express the same NSC markers as those in the SVZ, such as *Sox2*, *Sox9* and *CD133*. Multipotent ECs are largely quiescent, proliferate at low rates, and generate few astrocytes and oligodendrocytes in healthy conditions (Hachem et al., 2020), indicating that spinal cord tissue is by default pro-gliogenic. EAE activates ECs, that besides predominantly generating astrocytes, also form OPCs that subsequently differentiate into myelinating oligodendrocytes (Meletis et al., 2008). Systemic TH treatment counteracted a hypothyroid state in the spinal cord of EAE marmosets and facilitated remyelination (D’Intino et al., 2011), but whether or not that was by acting on ECs or by stimulating pOPCs to differentiate is unknown. Whether and how TH could act on ECs will be of interest for future research.

A final question in this context is that of the relative benefit of SVZ-OPCs over pOPCs. As already noted, SVZ-OPCs remyelinate axons with sheaths that have normal thickness (Xing et al., 2014; Brousse et al., 2015; Remaud et al., 2017), in contrast to otherwise thinner-than-normal sheaths produced by pOPCs. In addition, pOPCs isolated from adult rat brains had a reduced differentiation potential and remyelination capacity as compared to those of young brains (Neumann et al., 2019), making them a less attractive target in a disease that primarily affects older people. Remarkably, OPC generation in the SVZ and the rostral migratory stream remains stable throughout life, contrasting a sharp decrease in neurogenesis (Capilla-Gonzalez et al., 2013; Ernst et al., 2014; Weissleder et al., 2016). A mild hypofunction of the thyroid as observed in many elderly (Bowers et al., 2013; Biondi et al., 2019) might even favor SVZ-OPC genesis. The question remains though, if they are able to differentiate efficiently in aging conditions.

Further examination of TH-stimulated remyelination efficacy by SVZ- and EC-derived OPCs in MS models will be paramount to get an idea of the spatiotemporal white matter improvement, whether this process can be accelerated, and how long naked axons remain exposed to harming components of the extracellular matrix, which can potentially lead to irreversible neuronal and axonal degeneration (Franklin et al., 2012) as is the case in progressive MS (Michailidou et al., 2014). Intervening earlier might intensify recovery by endogenous repair mechanisms and increase chances of halting disease progression or even reverse damage. However, since plaque progression and demyelination occur heterogeneously, choosing an optimal therapeutic time window will be very complicated.

### Translational Aspects of Thyroid Hormone-Induced Remyelination

Given that (i) TH has consistent effects on oligodendrocyte differentiation and remyelination in animal models of MS, (ii) SVZ-oligodendrogenesis is maintained throughout human life (Ernst et al., 2014; Weissleder et al., 2016) and (iii) SVZ-OPC generation increases upon MS-induced injury in humans (Nait-Oumesmar et al., 2007), we and others propose that there is strong potential for TH as a treatment in MS patients. A clinical phase 1 trial (NCT02760056) recently confirmed short-term safety and tolerance for low systemic doses of synthetic T_3_ in a cohort of 15 MS patients (Wooliscroft et al., 2020) paving the way for a phase 2 trial. Sobetirome is currently in a phase 1 trial in patients suffering from the demyelinating disease X-linked adrenoleukodystrophy (NCT01787578). Safety and efficacy of such compounds will require testing in combination with anti-inflammatory agents currently used to control relapsing immune reactions. Further exploring underlying signaling pathways and mapping interaction with other factors interacting with NSCs and progenitor commitment (Akkermann et al., 2017), should lead to better treatment scenarios, where definitive promotion of the endogenous repair potential by T_3_ or CNS-specific TH analogs could pave the way to a reparative cure in MS and more largely in demyelinating diseases.

## The Repair Potential of Thyroid Hormone in Alzheimer’s Disease

Alzheimer’s disease is the most common cause of dementia, with a prevalence that tops of at 22.53% for individuals older than 85 years in Europe at 2016 (Niu et al., 2017). In AD, the accumulation of amyloid plaques and formation of neurofibrillary tangles trigger cerebral neuronal cell death and atrophy, causing progressive deterioration of cognitive functions and memory. An accelerated decrease in adult hippocampal neurogenesis (AHN) and hippocampal shrinkage are disease hallmarks in humans (Callen et al., 2001; Moreno-Jiménez et al., 2019) and compromise learning, memory and navigation (Tincer et al., 2016) as newly-generated granule cells normally rewire and strengthen connections in hippocampal circuits to promote learning- and memory-dependent plasticity. Most pharmacological approaches currently fail to reverse, halt or even slow down these symptoms. Counteracting both reduced AHN and neuronal cell loss could improve, or at least sustain memory processing (Tincer et al., 2016).

### The Link Between Thyroid Hormone, Hippocampal Neurogenesis and Alzheimer’s Disease

For years now, there have been controversial links between systemic TH status and the pace of human hippocampal neurogenesis. Adult-onset hypothyroidism, the second-most common human endocrine disorder, associates with depression, and shares the hallmarks of reduced AHN and decreased hippocampal volume with AD, as well as memory impairments and emotional alterations (Cooke et al., 2014; Planchez et al., 2020). T_4_ treatment in (sub)clinical hypothyroid patients reversed underperformance on several cognitive tests specifically designed for assessing hippocampal function (Correia et al., 2009). Of the 159 patients with bipolar disorder, and of whom none responded to a wide range of antidepressants, 84% had improved cognitive symptoms after supplementing supraphysiological T_3_ doses (Kelly and Lieberman, 2009). However, in this and other small-scale clinical studies, T_3_ and also T_4_ were always given as an adjuvant therapy when antidepressants fail, leaving the effect of THs alone untested (Parmentier and Sienaert, 2018). Interestingly, low T_3_ levels as well as DIO2 polymorphisms predispose for developing AD (McAninch et al., 2018; Quinlan et al., 2019) and T_3_ levels were reduced in brains of deceased AD patients (Davis et al., 2008), suggesting that hypothyroid conditions may affect AHN similarly as in depression.

Animal studies showed that TH regulators limit TH action to specific stages of hippocampal neurogenesis that lead to the production of new granule neurons in normal conditions, cells that will eventually rewire dentate gyrus circuits to contribute to hippocampal neuroplasticity. Adult-onset hypothyroidism and KO of *TR*α*1*, *Dio2* or *Mct8* in rodents showed that NSC proliferation was not affected, but that hippocampal neuroblast generation, differentiation and survival of newly generated neurons were decreased (Desouza et al., 2005; Kapoor et al., 2012; Bárez-López et al., 2014; Sánchez-Huerta et al., 2016; Mayerl et al., 2020). Systemic T_3_ treatment effectively recovered these processes and rescued neurological functions (Desouza et al., 2005; Eitan et al., 2010; Kapoor et al., 2010). It is tempting to speculate that TH can thus stimulate AHN to counteract hippocampal neuronal loss in AD too (Figure 2), as long as it can be started soon enough. So far, only few studies have assessed this potential in appropriate AD models or patients.

### Thyroid Hormone as a Repair Cue in Alzheimer’s Disease

The effect of TH treatment was tested in a mouse model whereby amyloid β, the main constituent of amyloid plaques in the AD brain, was injected in the hippocampus to mimic AD. Seven days later, a 4-day long daily intraperitoneal administration of low T_4_ doses rescued granule cell numbers, reduced hippocampal neuronal cell death, increased cholinergic function, and restored cognitive functions (Fu et al., 2010). Similarly, intraperitoneal administration of T_4_ to healthy 22–23-month-old rats stimulated *Ngf* and *Nt-3* expression in the hippocampus, reflecting neuroprotective TH actions, even in these relatively aged rats (Giordano et al., 1992). These data coincide with 3-month-old thyroidectomized rats that displayed reduced AHN and performed less well in a forced-swimming test, a measure for depressive-like behavior. Both a single intraperitoneal injection of T_4_ + T_3_ and a daily supply via the drinking water rescued neurogenesis and restored behavioral performance, suggesting rapid effects of TH on AHN (Montero-Pedrazuela et al., 2006).

Apart from stimulating neurogenesis and providing neuroprotection, TH could act on NSCs via another, more indirect way. Amyloid β was shown to accumulate in SGZ-NSCs in mouse AD models, inhibiting proliferation and increasing apoptosis, thus blocking AHN (Haughey et al., 2002b). T_3_ negatively regulates the amyloid-β precursor protein (*APP*) gene (Belandia et al., 1998; Belakavadi et al., 2011), and rendering rats hyperthyroid by giving oral T_3_ for 5 days strongly suppressed overall brain *APP* expression (Belakavadi et al., 2011). However, the sole study that tested this trait in a rat model for AD by giving supraphysiological doses of T_3_ for 4 weeks showed no significant changes in brain amyloid β content by western blotting (Prieto-Almeida et al., 2018) (Figure 2). T_3_ did reduce Tau hyper-phosphorylation and glycogen synthase kinase 3 α/β (GSK-3β) expression (Prieto-Almeida et al., 2018). Hyperactivity of each of these factors underlies AD pathogenesis and associates with amyloid β deposition, plaque forming and memory impairment (Hooper et al., 2008).

Amyloid β also reduces SVZ-NSC renewal capacity (Haughey et al., 2002a), corresponding to decreased numbers of proliferating stem cells in the SVZ of AD patients (Ziabreva et al., 2006; Rodríguez et al., 2009). As AD patients often suffer from anosmia (McShane, 2001), this might suggest impaired SVZ-neurogenesis, although the role of SVZ-neuroblasts in human olfaction is still under debate (Lim and Alvarez-Buylla, 2016). T_3_-dependent restoration of SVZ-neurogenesis could be a potential source of neurons to counteract cerebral atrophy, as the SVZ is the largest NSC reservoir. In *in vitro* neurospheres generated from SVZs dissected from rats in which streptozotocin injection replicated AD hallmarks, T_3_ administration recovered NSC proliferation and *Seladin-1* expression (Mokhtari et al., 2020), preventing caspase 3-mediated neuronal apoptosis (Peri and Serio, 2008).

TTR could represent another important factor in AD (Figure 2). TTR efficiently binds amyloid β in- and outside cells (Buxbaum et al., 2008; Li et al., 2011), preventing aggregation and toxicity in hippocampal neurons (Silva et al., 2017) and clearing amyloid β from the brain (Alemi et al., 2016). TTR levels in the brain and CSF have been reported to be decreased in several AD patients and could be involved in disease progression (Hansson et al., 2009). These reduced TTR levels, together with the possibility that TTR protein sequesters amyloid β, could reduce T_4_ availability (Li and Buxbaum, 2011) and hamper TH supply to SVZ-NSCs, affecting SVZ-neuro- and oligodendrogenesis. *Ttr* KO mice had decreased numbers of newly SVZ-generated neuroblasts and favored glial fate choice (Vancamp et al., 2019; Alshehri et al., 2020), reminiscent of the effects on SVZ-neurogliogenesis following hypothyroidism (López-Juárez et al., 2012; Remaud et al., 2017).

So far, only one study in human AD patients demonstrated that intravenous TRH injections improved memory and concentration (Mellow et al., 1989), probably through increased TH release by the thyroid gland, as patients experienced tachycardia, an indicator of hyperthyroidism. However, TRH could also have extra-hypothalamic effects, and directly regulate cellular processes associated with AHN. Reduced TRH levels were detected in the hippocampus of AD patients, and *in vitro* experiments with rat hippocampal neurons demonstrated that intracellular TRH depletion increased *GSK-3*β expression and Tau phosphorylation (Luo et al., 2002; Luo and Stopa, 2004). Hyperphosphorylated Tau can accumulate and have neurotoxic effects (Rajmohan and Reddy, 2017). In contrast, administrating TRH to neuronal cultures reduced *GSK-3*β expression and Tau phosphorylation, thereby increasing neuronal survival (Luo and Stopa, 2004). TRH can also directly modulate synaptic activity of hippocampal neurons (Zarif et al., 2016). Such non-classical functions indicate a cellular signaling role for TRH in neurodegenerative diseases (for a review, see Daimon et al., 2013), although the underlying mechanisms remain incompletely understood.

A potential pro-regenerative function of T_3_ or other TH signaling components in AD-accompanied (hippocampal) neurodegeneration still awaits more *proof-of-concepts*, but deserves further testing in appropriate models given their strong role in AHN in the brain, and the persistence of hippocampal neurogenesis in aged healthy and AD individuals (Moreno-Jiménez et al., 2019; Tobin et al., 2019). Ideally, the therapeutic potential is tested over long periods by administering TH in different phases of the disease to analyze long-term efficacy, reversibility of neurobehavioral hallmarks and potential side effects on other organs, determining the best treatment scenario and setting the base for clinical trials. Whether a TH treatment could ever replenish neurons in brain regions other than the hippocampus is less sure, notably given the strongly reduced neurogenesis elsewhere in the aged brain.

## Thyroid Hormone as a Repair Cue in Acute Traumatic CNS Injury

Almost every chronic neurodegenerative disease is accompanied by unique symptoms that progress via well-documented, protracted stages. Identical disease-underlying mechanisms provide the possibility of developing a uniform treatment strategy that is applicable before symptoms worsen. Acute CNS trauma on the contrary, causes instantaneous neuroglial cell death eradicating entire neural networks, with either a fatal outcome, or a profound impact on a patient’s psychomotor well-being depending on injury size, location and duration. Repair is extremely challenging as neural networks need to be rebuilt from scratch, demanding a harmonized interplay between neuro- and oligodendrogenesis, axonal damage repair, regrowth and reconnection of preserved axons, and myelinogenesis to functionally restore and reconnect CNS regions (O’Shea et al., 2017). Furthermore, acute trauma elicits an inflammatory response that besides initial wound healing sets in place a repair-blocking environment, one that is hostile for remnants of damaged neural networks. Stroke and acute SCI are two traumatic CNS events that must face each of these challenges.

### Thyroid Hormone Action on Neural Stem Cells in Stroke Injury

Stroke is a global leading cause of death and permanent disability, mostly affecting the elderly, and causing 5.5 million deaths 2016 alone (Gorelick, 2019). Stroke survivors often deal with permanent disabilities, including paralysis, sensory disturbances or impairments of higher cognitive functions such as language, functions that associate with regions in the cerebrum that are the most prone to stroke. Ischemic stroke occurs when a blood vessel in the brain is clogged, whereas a ruptured brain artery causes hemorrhagic stroke. A poor blood supply deprives brain tissue from oxygen and nutrients, and causes neuroglial apoptosis, forming an ischemic infarct region. NSCs that can potentially contribute to repair arise from regions next to the injury and from the stem cell zones, and rapidly respond to injury. Brain examinations of deceased stroke patients showed that capillary-associated pericytes convert into proliferative stem cells that express the NSC markers *Sox2* and *c-Myc*, with neuron-generating properties *in vitro* (Tatebayashi et al., 2017). Numbers of proliferative and neuronal marker-expressing cells also increased in the human ipsilateral SVZ after stroke (Marti-Fabregas et al., 2010).

Transgenic ablation of SVZ-derived DCX-positive neuroblasts 14 days prior to stroke induction in adult mice increased infarct size and worsened neurobehavioral disabilities 24 h after stroke (Jin et al., 2010), suggesting that enhanced SVZ-neurogenesis is a first-line repair response. The gliogenic response is however predominant as the SVZ output 2 weeks after stroke comprised 59% GFAP-positive astrocytes, 15–20% OPCs and only 10% DCX-positive neuroblasts, a distribution that almost did not change in the following weeks (Li et al., 2010). Tracing experiments demonstrated that SVZ-derived uncommitted precursors migrate to the damaged murine cortex, the first arriving within 4 days post-stroke, where most of them give rise to reactive astrocytes that form a glial scar (Faiz et al., 2015). Some of them can convert into neurons under forced expression of *Ascl1*, but poor cell survival in a hostile inflammatory environment as well as a lack of pro-regenerative cues prohibit recovery of damaged cellular networks (Xing et al., 2012; Lindvall and Kokaia, 2015) leaving many stroke victims disabled for life (Mitsios et al., 2007).

In humans, lower free T_3_ levels correlate with increased stroke severity, complications and poorer rehabilitation (Bunevicius et al., 2015; Lamba et al., 2018) suggesting that therapeutically restoring TH levels could enhance endogenous repair responses. The potential of TH as a repair cue has been tested in adult mice and rats wherein middle cerebral artery occlusion (MCAO) simulates permanent or transient stroke, the latter allowing re-perfusion of tissue 45 – 120 min after the occlusion. T_3_ injection either once 24 h after cerebral ischemia, or daily for 6 days, increased cell proliferation and expression of the neurogenic markers *Bdnf*, *Nestin* and *Sox2* in the SVZ 7 days later (Akhoundzadeh et al., 2017; Sabbaghziarani et al., 2017). Unfortunately, which cell types proliferated more actively was not investigated, nor was the impact on neuronal vs. glial specification. *Sox2* is negatively regulated by T_3_ under physiological conditions (López-Juárez et al., 2012) but might here simply reflect higher NSC numbers, as T_3_ and TRα1 also maintain full proliferative activity of SVZ-NSCs (Lemkine et al., 2005), or alternatively a reactivation of quiescent NSCs. Apart from the SVZ, focal cerebral ischemia in adult rats also stimulated hippocampal neurogenesis (Jin et al., 2001). A single intra-cerebroventricular dose of T_3_ right after transient MCAO in adult rats increased neuronal survival within the SGZ 3 weeks later through enhancing expression of the neurotrophic factors *Bdnf* and *Gdnf*, thereby improving memory and learning capabilities (Mokhtari et al., 2017). A single T_4_ dose 1 h after traumatic brain injury in rats also induced *Dcx* in the hippocampus within 24 h (Li et al., 2017). This suggests that stroke occurring in distant brain regions such as the cortex can influence AHN and associated memory functions, and that TH can stimulate SGZ neurogenesis and hippocampal circuit formation during the recovery phase.

Interestingly, *Ttr* mRNA transcripts also increased in various hippocampal cell types (e.g., ependymal cells, microglia and mature neurons) 24 h after traumatic brain injury in adult mice. Although expression of membrane TH transporters did not change, the authors speculated that this increase reflected cellular hypothyroidism, and subsequently tested neurobehavioral recovery after giving a single intraperitoneal dose of T_4_ immediately after the injury. One week later, using a single-cell genomic approach, the authors observed that the dysregulated expression of 93 out of 121 genes linked to cellular metabolism and hormone response pathways in hippocampal cells, including *Ttr*, was restored, and that mice performed better on memory tests (Arneson et al., 2018). While TH promotion of SGZ-neurogenesis can thus improve hippocampal-related functions, new neuroblasts probably do not migrate away from their site of origin (Ibrahim et al., 2016), and hold less promise for repair at the actual site of injury in comparison to more efficiently migrating SVZ-OPCs and neuroblasts, at least in rodents (Flygt et al., 2013; Faiz et al., 2015).

New-born cells can be vulnerable to pro-apoptotic proteins that are found in injured gray matter in humans (Mitsios et al., 2007). Injection of a plasmid expressing the anti-apoptotic BCL2 protein in the lateral ventricles of rats following MCAO, protected neural progenitor cells and new-born mature neurons in the striatum from cell death (Zhang et al., 2006). Being a well-known TH-responsive gene (Morris et al., 2017), TH administration successfully reduced apoptosis 24 h after injury by inducing *Bcl2* expression (Crupi et al., 2013; Genovese et al., 2013). Additionally, all these studies consistently found that post-stroke TH administration additionally preserves tissue integrity and has important anti-inflammatory effects, curtailing infarct volume and shaping a pro-survival environment in which improved synaptic plasticity enhances neural network recovery and mitigates post-injury behavioral functions (Crupi et al., 2013; Genovese et al., 2013; Akhoundzadeh et al., 2017; Li et al., 2017; Mokhtari et al., 2017; Rastogi et al., 2018; Talhada et al., 2019).

There are currently no data to which extent TH treatment repairs axonal, oligodendrocyte and myelin loss after stroke, and it has yet to be explored which local regulators of TH action could mediate effects in neurogenic zones. Certainly, governing NSC fate choice will be difficult requiring exact timing protocols. In addition, whether newly formed neurons are able to span the greater distances of the human brain is unknown (discussed in Nemirovich-Danchenko and Khodanovich, 2019). A diminished regenerative capacity during aging due to declining NSC pool sizes (Villeda et al., 2011) is another important issue as the elderly are especially prone to be stroke victims. Old mice recover less well than their younger counterparts due to exacerbated immune responses, data reflected by a higher neutrophil infiltration and infract size in aged *post-mortem* human stroke brains too (Ritzel et al., 2018). Most experimental studies investigating the effects of TH used young, 2–3 month-old animals, which may not represent the physiology of an older human being, notably with regard to the sharp drop in SVZ-neurogenesis and overall diminished TH levels during aging. One study demonstrated that the beneficial effects of T_3_ combined with bone marrow stem cell transplantation and mild exercise in 2-month-old mice on stroke outcome (Akhoundzadeh et al., 2017) were not replicated in 12-month-old mice (Akhoundzadeh et al., 2019). Thus, it is preferable that future experiments examine how NSCs in young and old animals respond to ischemic injury in parallel, hence acquiring a more reliable image of the potential therapeutic effects of TH. Lastly, determining the right timing, dose and administration route will be imperative, as subtle shifts in TH homeostasis, for instance due to ESS, can provoke adverse effects on stroke recovery. Infarct size and vascular integrity aggravated after MCAO-induced stroke in hyperthyroid rats (Keshavarz and Dehghani, 2017), and the T_4_ treatment that ameliorated dysregulated gene expression in hippocampal cells after injury in the mice from the study of Arneson et al., also changed non injury-related genes that are associated with pathways key for proper brain function (Arneson et al., 2018). Subclinical hyperthyroid stroke patients also had increased neurological disability and a higher risk of recurrences (Wollenweber et al., 2013). Each and every one of these issues needs to be thoroughly addressed to obtain a more comprehensive, systems biology picture of which TH treatment procedure could eventually comply with optimal injury repair.

### Thyroid Hormone in Stem Cell Repair After Traumatic Spinal Cord Injury

Spinal cord trauma directly disrupts the bidirectional information flow between the brain and peripheral effectors, risking the loss of sensation and voluntary movement of tissues innervated by neurons below the site of injury. An estimated 27 million people worldwide suffer from a form of irreversible motor disability caused by SCI (James et al., 2019). As in stroke, neurons and glia die upon SCI injury and an exacerbated inflammatory and molecular inhibitory environment constrains repair (Rust and Kaiser, 2017). Endogenous NSCs could replace cells, but cannot be mobilized from distant brain stem cell zones except if they are transplanted into the lesion site (e.g., Vismara et al., 2017; Sankavaram et al., 2019). Endogenous repair could be however be stimulated by engaging the spinal cord ECs. Upon SCI, they proliferate, migrate and differentiate into astrocytes, forming a tissue scar in the first instance, and also less frequently OPCs (Meletis et al., 2008; Moreno-Manzano et al., 2009; Cawsey et al., 2015). Neurogenesis in the spinal cord is essentially non-existent in normal or injured conditions (Meletis et al., 2008), but ECs have neurogenic potential when transplanted in the SVZ (Shihabuddin et al., 2000) as well as *in vitro* after adding factors that stimulate neuronal differentiation (Moreno-Manzano et al., 2009). Modulating EC fate and boosting multi-lineage cell generation and differentiation could thus facilitate SCI repair.

The beneficial effects of THs on OPC differentiation as observed in EAE-induced spinal cord demyelination in rodent models of MS (Calza et al., 2002; Dell’Acqua et al., 2012), could also initiate myelin repair in SCI, although EAE does not reflect the loss of all cell types. A combination of T_4_ pulses with valproic acid right after disease onset improved clinical signs after EAE-induced demyelination in adult rat spinal cords, but did neither increase OPC numbers nor improve myelin status (Castelo-Branco et al., 2014) (Table 1). Continuous intraperitoneal T_4_ administration following transplantation of Nestin-positive NSCs, derived from embryonic stem cells, into the injury site 10 days after SCI in mice, did not improve coordinated motor behavior more than the stem cells grafts alone (Kimura et al., 2005). Whether or not manipulating local TH signaling could allow directing EC fate to stimulate neurogenesis remains unknown, and primary experiments should aim to determine how T_3_ affects cell fate choice in physiological conditions. Only one study demonstrated that local administration of a hydroxy gel containing T_3_ on the injury site caused by cervical contusion successfully increased oligodendrocytes and improved myelination (Shultz et al., 2017). ECs also express *Sox2*, which is a T_3_-responsive cell fate switch in SVZ-NSCs (López-Juárez et al., 2012). However, in contrast to repressing *Sox2* in favor of SVZ neuroblast generation, stimulating *Sox2* reprogrammed reactive astrocytes to form proliferative DCX-positive neuroblasts in mouse SCI, which further matured into synapsing interneurons when accompanied by valproic acid (Su et al., 2014). Also in adult zebrafish SCI, increased *Sox2* expression stimulated EC proliferation and the formation of new motor neurons resulting in functional repair (Ogai et al., 2014). Another potential target might be *Egfr*, shown to trigger EC proliferation in SCI (Liu et al., 2019). Since *Egfr* is a gliogenic factor in SVZ-NSCs that is negatively regulated by T_3_ (Remaud et al., 2017), TH treatment could promote neuronal determination. Further research into cell-type specific expression patterns of TH regulators in both healthy- and disease-based contexts might uncover which cell types respond to TH in SCI (Boelen et al., 2009; D’Intino et al., 2011; Fernández et al., 2016).

## Future Perspectives on Thyroid Hormone-Mediated CNS Repair

Endogenous NSCs are activated notably upon injury in the adult human CNS, but the NSC progeny fails to replace neurons and glial cells due to molecular inhibition and the lack of pro-regenerative cues, leaving most victims of CNS injury disabled for life. Pre-clinical studies emphasize that TH, as a key regulator of NSC fate and progenitor behavior in physiological conditions, has the potential to override the blocked endogenous repair response in various pathophysiological contexts, despite the different etiologies of disease. TH effectively regulates oligodendrogenesis and remyelination in models of MS, and while showing some beneficial actions in other CNS insults, future experiments have to elucidate how TH can stimulate endogenous repair pathways in the SVZ, SGZ and the spinal cord.

### From Systemic to Local and Target-Directed TH Repair

A forthcoming challenge in TH-promoted CNS repair is to maximize and extend beneficial effects on NSCs and their progenitors while minimizing adverse effects on non-target tissues. The relative impact of parameters such as the dose, delivery route and treatment window will require scrupulous evaluation. For instance, the risk for developing (subclinical) hyperthyroidism increases with protracted TH treatment due to thyroid axis dyshomeostasis, or because disease progression or recovery enters a new phase that coincides with discrete alterations in the body’s hormone balance. Common side effects include cardiac arrhythmias and secondary osteoporosis (Flynn et al., 2010). Future pre-clinical studies applying systemic TH treatments to CNS injury models should therefore test therapeutic efficacy over longer periods while simultaneously monitoring TH serum levels and peripheral organ physiology.

A promising alternative are the TH analogs (e.g., Triac, Ditpa, sobetirome) that mimic TH action, but have CNS- or even cell type-selective actions because of TR isoform preference. Another, so far less explored way, is providing TH or TH analogs to stem cell zones by alternative routes, for instance via the cerebroventricular system (Bárez-López et al., 2019), using nanoparticles (Mdzinarishvili et al., 2013), or even via direct polyethylenimine-mediated NSC transfection (Lemkine et al., 2002). The benefit of such an approach is that TH signaling pathways relevant for a repair process could be directly manipulated in target brain regions or cell populations while leaving neighboring regions unscathed. The administration route can thereby be chosen in function of the disease or injury. Individuals suffering from the Allan-Herndon-Dudley Syndrome provide a clear example of the power such approach can have. Patients are permanently intellectually disabled due to severe cerebral hypothyroidism caused by a mutated MCT8, but have elevated serum TH levels, eliminating the option of systemic TH treatment. However, a brain implant safely delivered the TH analog Triac into the cerebral ventricles of *Mct8* KO mice increasing its content in the brain, without worsening cerebral hypothyroidism nor peripheral thyrotoxicosis (Bárez-López et al., 2019). In the field of nanomedicine, NFL protein-coated lipid nanocapsules that specifically interact with NSC proteins were successfully taken up by rat NSCs after intra-cerebroventricular injection (Carradori et al., 2016). When loaded with retinoic acid, they successfully induced oligodendrogenesis after lysolecithin-induced focal demyelination of the corpus callosum (Carradori et al., 2020). These *state-of-the-art* methods remain to be thoroughly tested in TH-mediated CNS repair, but will definitely revolutionize neuroregenerative medicine.

Currently, we have only limited evidence of which cell types and subpopulations are more preferentially affected by systemic and local TH treatments in CNS injury models. While a beneficial effect was observed on a given cell type, for example, in the case of the OPCs in MS models, the impact on other cell types was often not evaluated. However, given the widespread expression of TRs (Bernal, 2007), one can assume that possibly all cells are affected to some extent by a TH treatment. Regulators of TH availability that confine TH action in a subset of neurogenic niche cells could represent novel ways to manipulate TH signaling tissue- or cell type-specifically, but only if we comprehend their temporal-spatial expression patterns and function in both health and disease. This goes hand in hand with dissecting how the (epi)genetic NSC landscape changes during disease to identify which TH target genes are key in repair. Such knowledge will have to be considered in light of emerging data showing that heterogeneous NSC populations in distinct SVZ microdomains contribute to specific neuronal and glial subpopulations, and thus may have varying repair potential (Chaker et al., 2016; Akkermann et al., 2017; Mizrak et al., 2019).

### Other Future Aspects in TH-Mediated CNS Repair

Most studies that explored the repair potential of TH so far have been limited to rodent species in which NSC biology differs extensively from humans (Williamson and Lyons, 2018; Oppenheim, 2019). For instance, in contrast to mice, the generation of new neuroblasts is practically non-existent in the adult human SVZ and a higher percentage of NSCs is quiescent, whereas SVZ-oligodendrogenesis remains stable in both species (Sanai et al., 2004; Capilla-Gonzalez et al., 2013; Ernst et al., 2014). Such species-unique traits can be expected to partly explain why NSC-based therapies often lead to better functional outcomes in CNS injury in rodents as compared to humans. Use of non-human primate models can fill this evolutionary gap and help to estimate better to what extent beneficial effects of TH treatment on CNS repair will be replicated in humans (Tsintou et al., 2020). For example, the mouse lemur (*Microcebus murinus*) is a rapidly aging non-human primate, with 20% of the population developing aging-associated neurodegeneration at the age of 5 years, sharing many biological and behavioral characteristics with human AD, allowing to test disease progression and evaluate therapies better (Bons et al., 2006). On the other hand, comparative studies on the regenerative potential of TH in several non-mammalian vertebrates (Vanhunsel et al., 2020), deciphering which pathways enable successful repair, can give a better idea as to where, when and why they fail in mammals. Furthermore, most pre-clinical studies involving TH were performed on young animals, while many neurodegenerative diseases strike at older ages. It is not well understood how TH action on NSC processes such as fate choice changes during aging, and thus whether the potentially beneficial actions on CNS injury are fully preserved in the elderly. Such knowledge gaps emphasize the pertinence of fundamental research in these domains.

In addition, one cannot discuss TH signaling without considering other important, interconnected factors that regulate NSC behavior in normal and pathological conditions (see for instance Obernier and Alvarez-Buylla, 2019). In MS for example, Notch, Wnt and retinoid signaling, as well as the transmembrane signaling protein LINGO-1 also regulate oligodendrogenesis and thus represent additional therapeutic targets (Huang and Franklin, 2011). TH often interacts with such signaling pathways as well as with other hormones that influence neurogliogenesis (e.g., progesterone, testosterone, estrogen) (Hartley et al., 2014), demanding an integrative systems biology approach to understand the effects of TH-replacing therapy fully.

Lastly, in the case of chronic neurodegenerative diseases, many questions also remain with regard to the pathophysiological mechanisms or origins that lead to disease in the first place, that underlie disease progression, or that account for unexplained incidence trends and gender-specific effects. In MS for example, an unexplained global shift of 2/1 to 3/1 in the ratio of women versus men occurred over the past decades (Reich et al., 2018; Wallin et al., 2019). Recent evidence suggests that MS is at least partially provoked or exacerbated by environmental factors (Leray et al., 2016; Bove, 2018). Since MS incidence is considerably high in industrialized countries, one possibility is that endocrine disrupting chemicals (EDCs) to which we are daily exposed are implicated. Chemical production has increased more than 300-fold since 1970 (Demeneix, 2019), and many of them share the halogenated structure with THs, making them potentially able to provoke adverse, disrupting actions on TH signaling pathways (Boas et al., 2012). For several EDCs, it has already been shown they affect adult stem cell self-renewal, lineage decisions and differentiation programs (Kopras et al., 2014). As shown in *post mortem* brain samples, lipo-soluble EDCs also accumulate in myelin, in essence a stack of lipid sheaths (van der Meer et al., 2017) and could thus have toxic effects that trigger oligodendrocyte apoptosis. Developmental exposure could also compromise NSC repair potential in later life through epigenetic misprogramming (Jacobs et al., 2017). There is an urge to elucidate whether or not early-life EDC exposure renders the brain vulnerable to diseases or malfunctions later in life (Porta and Vandenberg, 2019), since developmentally disrupted TH signaling has already been linked to increased risk for autism spectrum disorder, attention deficit/hyperactivity disorder and schizophrenia (Modesto et al., 2015; Gyllenberg et al., 2016).

In conclusion, improved understanding and new established concepts in TH physiology at the cellular and molecular level, are opening up new avenues for boosting NSC-dependent repair with TH as a pro-repair cue. Clinical trials could lead to therapies that might one day facilitate reversing currently untreatable disabilities that accompany CNS injury.

## Author Contributions

PV and SR worked out the concept of the manuscript. PV made the figures and table. PV, LB, BD, and SR wrote the manuscript and all authors verified the final version of the manuscript.

## Conflict of Interest

The authors declare that the research was conducted in the absence of any commercial or financial relationships that could be construed as a potential conflict of interest.

## Abbreviations

AD: Alzheimer’s disease
AHN: adult hippocampal neurogenesis
APP: amyloid precursor protein
BBB: blood-brain barrier
BCSFB: blood-cerebrospinal fluid barrier
CNS: central nervous system
CSF: cerebrospinal fluid
DIO: deiodinase
EAE: experimental autoimmune encephalomyelitis
ECs: ependymal cells
EDCs: endocrine disrupting chemicals
ESS: euthyroid sick syndrome
GSK-3 α / β: glycogen synthase kinase 3 α / β
HPT: hypothalamus-pituitary-thyroid
IHC: immunohistochemistry
LAT: large neutral amino acid transporter
MBP: myelin basic protein
MCAO: middle cerebral artery occlusion
MCT: monocarboyxlate transporter
MRI: magnetic resonance imaging
MS: multiple sclerosis
NSC: neural stem cell
OATP1C1: organic anion transporting polypeptide 1C1
OPC: oligodendrocyte precursor cell
pOPC: parenchymal oligodendrocyte precursor cell
rT_3_: reverse T_3_ or 3,3 ′,5 ′ -triiodothyronine
SCI: spinal cord injury
SGZ: subgranular zone
SVZ: subventricular zone
T_3_: 3,5,3 ′ -triiodothyronine
T_4_: 3,5,3 ′,5 ′ -tetraiodothyronine or thyroxine
TAP: transient amplifying progenitor
TH: thyroid hormone
TR: thyroid hormone receptor
TRH: thyrotropin-releasing hormone
TSH: thyroid-stimulating hormone or thyrotropin
TTR: transthyretin.
